# A review of mental health recovery programs in selected industrialized countries

**DOI:** 10.1186/s13033-016-0104-4

**Published:** 2016-12-01

**Authors:** Harold A. Pincus, Brigitta Spaeth-Rublee, Grant Sara, Elliot M. Goldner, Pamela N. Prince, Parashar Ramanuj, Wolfgang Gaebel, Jürgen Zielasek, Isabell Großimlinghaus, Margo Wrigley, Jaap van Weeghel, Mark Smith, Torleif Ruud, John R. Mitchell, Lisa Patton

**Affiliations:** 1 Department of Psychiatry, Columbia University, 1051 Riverside Drive, Room 5813, Unit 09, New York, NY 10032 USA; 2 Irving Institute for Clinical and Translational Research, Columbia University and New York–Presbyterian Hospital, New York, NY USA; 3 RAND Corporation, Pittsburgh, PA USA; 4 Mental Health Services and Policy Research, New York Psychiatric Institute, New York, NY USA; 5 InforMH, Health System Information and Performance Reporting Branch, NSW Ministry of Heath, Sydney, Australia; 6 Discipline of Psychiatry, Sydney Medical School, University of Sydney, Sydney, Australia; 7 Faculty of Health Sciences, Centre for Applied Research in Mental Health & Addiction (CARMHA), Simon Fraser University, Burnaby, BC Canada; 8 Royal Ottawa Health Care Group, Ottawa, ON Canada; 9 Division of Mental Health Services and Policy Research, Columbia University Medical Center, New York, NY USA; 10 Department of Psychiatry and Psychotherapy, Medical Faculty, Heinrich Heine University Düsseldorf, Düsseldorf, Germany; 11 The LVR Institute of Healthcare Research, Düsseldorf, Germany; 12 The WHO Collaborating Center for Quality Assurance and Empowerment of Mental Health, Düsseldorf, Germany; 13 Mental Health Services, Dublin, Ireland; 14 Phrenos Centre of expertise, Utrecht, The Netherlands; 15 Tilburg School of Social and Behavioral Sciences, Tranzo Scientific Centre for Care and Welfare, Tilburg University, Tilburg, The Netherlands; 16 Te Pou, National Centre of Evidence Based Workforce Development for the Mental Health, Addiction and Disability Sectors, Auckland, New Zealand; 17 Division Mental Health Services, Akershus University Hospital, Lørenskog, Norway; 18 Institute of Clinical Medicine, University of Oslo, Oslo, Norway; 19 Mental Health and Protection of Rights Division, Population Health Improvement Directorate, The Scottish Government, St Andrew’s House, Edinburgh, Scotland, UK; 20 Division of Evaluation, Analysis and Quality, Center for Behavioral Health Statistics and Quality, SAMHSA, Rockville, USA

**Keywords:** Mental health, Recovery measurement programs, Quality

## Abstract

The concept of recovery has gained increasing attention and many mental health systems have taken steps to move towards more recovery oriented practice and service structures. This article represents a description of current recovery-oriented programs in participating countries including recovery measurement tools. Although there is growing acceptance that recovery needs to be one of the key domains of quality in mental health care, the implementation and delivery of recovery oriented services and corresponding evaluation strategies as an integral part of mental health care have been lacking.

## Background

The recovery movement has attracted widespread interest over the last decade, and as a result, has become part of broader change and improvement processes across mental health systems in many industrialized countries. The concept of recovery emerged from the service user movements in the 1970s, most notably in Anglo-Saxon countries, challenging traditional medical approaches to treating people with mental illness and how services for these individuals are organized and delivered. At the core of this movement is the shift from services based on the clinical meaning of recovery (i.e., treatment and symptom reduction as manifested by clinical assessment tools such as the PHQ-9) to recovery as defined by the service user’s view of what is needed or desirable in the care s/he is encountering to help him/her resume a meaningful life and valued roles.

One of the earliest definitions of recovery refers to “a deeply personal, unique process of changing one’s attitudes, values, feelings, goals, skills and/or roles… a way of living a satisfying, hopeful and contributing life even with the limitations caused by illness” [1]. Person orientation and person involvement are some of the guiding principles of recovery oriented practice; however, common understanding of underlying concepts of recovery and implications of a recovery orientation of services is still emerging. Leamy et al. [2] developed an empirically based conceptual framework of recovery centered around connectedness, hope and optimism about the future, identity, meaning in life, and empowerment (CHIME). Against this background, clinical experts of the International Initiative for Mental Health Leadership (IIMHL) initiated a study to examine the current status of recovery-oriented practices and measurement activities across ten countries. The group, under the auspice of the IIMHL, initiated an international project, “Measuring Quality of Mental Health Care: An International Comparison” in 2008. This ongoing initiative aims to raise awareness among clinicians and policymakers regarding the quality of care of their respective mental health systems, and ultimately to be able to compare system performance across countries to inform initiatives for transformation of mental health services.

Results of an international literature review in Phase I of this project found the recovery domain to have among the fewest measurable indicators of all domains in our sample [3]. Another study based on a survey of IIMHL clinical leaders in participating countries showed that few countries have successfully incorporated recovery measures into their national mental health quality measurement programs [4]. This points not only to a broader gap with regard to recovery measurement but the development and operationalization of recovery concepts and the integration of recovery in the broader arena in general. Building on work of Phase I and applying a modified Delphi process, Phase II of the study focused on developing consensus for a core set of performance and outcomes measures that could potentially be collected by all participating countries [5]. However, due to the underrepresentation of recovery measures within the overall portfolio of outcomes measures in participating countries, recovery measures were not part of the Delphi process and further consideration for inclusion into the core set of performance and outcomes measures.

Recognizing this gap, Phase III of the IIMHL study included a separate study component to examine the current status of recovery-oriented measurement activities and tools and to develop a phased strategy for enhancing the development of recovery-oriented measures for quality improvement and accountability across countries.

## Methods

We asked country lead representatives of participating countries to consult and review information on recovery-oriented activities and programs currently or soon to be implemented in their respective countries that might not be documented in existing literature (peer-reviewed or gray literature). In addition, country leaders were asked to identify and review peer-reviewed journal articles and gray literature that concern recovery-oriented instruments and measurement tools in use or under consideration within their countries to advance a recovery orientation in mental health services and systems. We deliberately applied an open-ended approach to inclusion of information on recovery without providing a specific definition of recovery to acknowledge not only the broad variety of existing definitions of recovery and its underlying concepts but also the different degree to which recovery principles have penetrated the delivery of mental health services within participating countries.

## Results—summary of recovery programs and initiatives across participating countries

The section below provides a brief overview of major programs and initiatives in each of the participating countries.

### Australia

Recovery has been an important priority in Australia’s national and state mental health policies, [6–8] service standards, [9] and workforce standards [10]. In 2013, the Australian Health Ministers’ Advisory Council released the *National Framework for recovery*-*oriented mental services* [11] and an accompanying guide for practitioners and providers [12]. The framework describes five ‘practice domains’: (1) Promoting a culture of hope and optimism, (2) Person first and holistic, (3) Supporting personal recovery, (4) Organisational commitment, and (5) Action on social inclusion and social determinants. These are supported by 17 ‘key capabilities’, including core principles, values, knowledge, attitudes, behaviors, skills and abilities.

To support measurement efforts, the Australian government funded a review of recovery measures suitable for routine use in the Australian context. The review [13] identified eight potentially suitable instruments: four designed to measure individuals’ recovery (recovery assessment scale; illness management and recovery scales; stages of recovery instrument; recovery process inventory) and four designed to assess the recovery orientation of services (recovery oriented systems measure; recovery self assessment; recovery oriented practices index; recovery promotion fidelity scale). However, no single measure was identified that met all clinical and policy requirements. In response, Australia’s national information committee has since developed two recovery-informed measures for potential national implementation. A measure of service user experience of care (the your experience of service questionnaire) [14] has been developed based on recovery principles within the National Standards for Mental Health Services, and is currently being implemented by several Australian states and territories. The ‘Living in the Community Questionnaire’ has been developed to measure social inclusion aspects of recovery [15].

### Canada

The Mental Health Commission of Canada (MHCC) is undertaking a two-phase initiative for the development and implementation of recovery guidelines building on Canadian and international models. This initiative concurs with the Commission’s development of an overall mental health strategy for Canada as well as other provincial/territorial activities in this area. To move toward a recovery-oriented mental health system, MHCC is proposing a framework that aims to align concepts of recovery (conceptual alignment) with necessary practice shifts (practical alignment) and wider contextual system transformations (contextual alignment) [16].

Many jurisdictions in Canada are incorporating the principles of recovery into mental health services. Initiatives at the state/territorial level include the *Healthy Mind, Healthy People* initiative from 2010 to address mental health and substance use in British Columbia [17]. This 10-year plan promotes a recovery approach across the entire spectrum of patient population groups from individuals with mild and moderate to severe and complex mental health and substance use problems and describes ambitious milestones within the set timeframe to transform the mental health system. Within the province of Ontario, the regions of Waterloo and Wellington-Dufferin have developed and implemented a framework based on an innovative partnership between mental health agencies and service user organizations to apply principles of recovery to system-wide case management [18]. The MHCC released a series of 55 national mental health indicators based on existing data sources, such as large-scale national epidemiological surveys [19]. Four of the indicators sought to report information related to recovery and the findings of each of these are summarized in Table 1 with full technical information is provided in a separate report [20].

### England

The ‘recovery approach’ has influenced English mental health policy both structurally and culturally. Recovery ideas have been the guiding vision of government policy since 2001 [21] and the recovery approach has increasingly shaped health strategy so that social inclusion and service user involvement are now core features of all mental health policy [22].

Focus has shifted towards the implementation and assessment of recovery principles [23]. In 2009 the government paper New Horizons [24] stated that “The effectiveness and acceptability of services will be assessed […] against indicators agreed between individual clinicians and service users, and used to help the service user plan their next steps towards recovery […]. Recovery-based services will ensure that people […] will have opportunities to take part in meaningful activities and to contribute to and participate in society”.

To assist in the delivery of these objectives, the Department of Health established the Implementing Recovery through Organisational Change [25] initiative in 2010 to assist mental health providers in establishing recovery-focused services. Concomitantly, the Joint Commissioning Panel for Mental Health issued guidance [26] to those commissioning such services on ‘Values based principles’ so that those with lived experience of mental illness are placed at the heart of any commissioning process and given ‘an equal footing to everyone else’.

In going forward, parity in health, [27] in which individuals with mental illness are afforded the same esteem and opportunities as those with physical illness, is seen as a key organizing principle in the drive towards more recovery-focused care and an implementation framework, the “No Health Without Mental Health Dashboard” [28] has been developed. While not a mental health recovery framework or measurement tool per se, many of the outcomes overlap with recovery-oriented measures such as: s*elf*-*reported well*-*being, employment, accommodation, quality of life, patient experience, self*-*management, confidence in challenging stigma/discrimination and overall satisfaction.*

### Germany

The German Association for Psychiatry, Psychotherapy and Psychosomatics (Deutsche Gesellschaft für Psychiatrie und Psychotherapie, Psychosomatik und Nervenheilkunde; DGPPN) develops evidence-based guidelines to facilitate diagnostic and treatment decisions between mental health providers and service users for specific diagnoses. These guidelines are based on the latest scientific evidence and are part of the national guidelines program issued by the German Association of the Scientific Medical Societies (AWMF). For example, the DGPPN treatment guideline on psychosocial treatments addresses the issue of recovery [29]. Recovery is defined as a process and consists of various components such as *hope, social inclusion, self*-*determination, quality of life, overcoming stigmatization, and empowerment*. An important goal of recovery is remission, using time and symptom based criteria (e.g., certain key symptoms should remain below a targeted threshold for 6 months and 2 years, respectively). Another important aspect of recovery is the strengthening of resilience.

Moreover, the DGPPN guideline on schizophrenia is currently being updated and transformed into a German Disease Management Guideline focusing on the coordination of care [30]. Evidence will be reviewed against the background of clinical (psychopathological) outcomes and recovery-oriented outcomes, such as quality of life and social and personal functioning of persons with schizophrenia. Another goal is the supplementation of the guideline with corresponding quality indicators.

### Ireland

In 2006, the Department of Health and Children recommended that Irish mental health services adopt a recovery perspective. The Health Research Board (HRB), the lead agency supporting and funding health research in Ireland, conducted a study to develop a coherent theory of recovery from mental health problems from the point of view of those recovering. Finalized in 2010, this study informs the Irish public about the possibility of recovery and the important role of community and is recommended for mental health professionals and educators, service users, carers, researchers, policymakers and the general public [31].

In addition, the department of health and children is supporting a number of Recovery based initiatives through their Genio Innovation fund, such as the advancing recovery in Ireland (ARI) project [32]. This is an 18-month initiative, which will support six mental health services in their efforts to implement a number of the key concepts in “A Vision for Change.” ARI focuses on service level structures, systems and practices that can maximize personal recovery opportunities and outcomes for service users. Each site will also introduce the “Recovery Context Inventory” tool (web-based mental health recovery profiling and outcome measurement tool). Currently seven sites are being evaluated and compared with adjacent mental health services which are not part of an ARI project to identify benefits of the ARI approach and guide wider implementation. Another Genio funded recovery project, EOLAS (“knowledge”), is now part of the Health Service Executive Programme for Mental Health and available nationally. The aim is to engage service users and their families in understanding the recovery journey following a diagnosis of a severe mental illness (SMI). It is an 8 week mental health information and learning programme co-facilitated by a peer as well as a clinical facilitator. Evaluation results showed that 84 % of participants with SMI reported improved well-being with significant impact on psychosocial outcomes.

### The Netherlands

To date, the concept of recovery overall has not had a strong impact on mental health practices and services directed at people with common mental disorders. However, recovery-oriented care is becoming more and more implemented in the care for people with SMI.

Recently a task force, by order of the Dutch minister of Health, developed *Crossing the bridge,* a national action plan to improve care for people with SMI, [33] in which recovery, empowerment, community integration and combating stigma are the key concepts. The overall ambition of this action plan is to help people with SMI help catch up with the rest of society and therefore ‘to improve their recovery (of health, participation and personal identity) by at least one-third in 2015’. To achieve this, the medical and support services should be organized in strongly coordinated local networks of mental health practitioners, service users and their families, supported housing agencies, general practitioners, vocational rehabilitation organizations, and generic social work teams on the basis of a national care standard and quality framework. This is to ensure that state-of-the-art treatment can be accessed by all people in the target group, regardless of their current position in the care landscape. Also local recovery colleges will be established that provide recovery courses, self-help services, and offer support in social inclusion issues.

Routine outcome monitoring of the key recovery dimensions is not yet mandatory; however, many Dutch mental health organizations are using the Manchester Short Assessment of Quality of Life (MANSA) instrument to measure the subjective quality of life of individuals with severe mental illnesses on a voluntary basis. Recently, a distinct Dutch Personal Recovery Scale was developed with the aim to have it added as a mandatory component of routine outcome monitoring in the Netherlands. Other instruments used for voluntary assessment in routine mental health care are the recovery oriented practices index (ROPI), the Quality Indicator for Rehabilitative Care (QuIRC), [34] and QUARTS (quality assessment of regional treatment systems for schizophrenia) [35]. Recently a group of Dutch practitioners and researchers became interested in the Individual Recovery Outcomes Counter tool (I.ROC), which has been developed by Penumbra, a third sector voluntary organization in Scotland. The Dutch translation of I.ROC will soon be piloted and validated in various mental healthcare practices in the Netherlands.

### New Zealand

In 1998, the New Zealand Mental Health Commission articulated in its initial *Blueprint* [36] strategy paper the need to move toward recovery-oriented mental health services. A second report *Blueprint II*—*Improving mental health and wellbeing for all New Zealanders*, published in 2012, builds on the first Blueprint document and is based on the concepts of people-centered and people-directed recovery and resiliency as core values of mental health services. Blueprint II extends the focus beyond the most severely affected individuals to those who have a lower level of need but whose mental health and addiction issues still have significant impact on their overall health and their ability to function at home or at work.

In 2012, The Ministry of Health [37] released its strategic development plan for mental health and addiction services (Rising to the Challenge) which views recovery as a guiding principle for services. Mental health services in New Zealand have been collecting and using outcome measurement tools for 8 years [38]. In the recently released New Zealand Health Strategy outcomes, measurement and indicators are stressed repeatedly [39].

Starting July 1 2015, New Zealand has mandated the collection of two recovery questions for its addiction services. The questions are part of an alcohol and drug outcome measure (ADOM) which consists of three sections: [40] frequency of drug and alcohol use; psychosocial impact of drug and alcohol use; and recovery. The two questions have become part of the national collection in New Zealand which means that New Zealand can report on this information at team, service and national levels. As a self reported outcome tool the two recovery questions provide an indication of where service users see their own recovery:

1. “Overall how close are you to where you want to be in your recovery?” (Tick the number that best fits where you are now 1, 2, 3, 4, 5, 6, 7, 8, 9, 10).
2. “How satisfied are you with your progress towards achieving your recovery goals?” (Not at all, slightly, moderately, considerably, extremely).

These two questions have recently been validated in terms of their psychometric properties [41, 42].

There are measures in use among certain provider organizations or patient sub-groups which explicitly address individual recovery, for example the Recovery Star is a widely used model, particularly in non-government organization settings. There is currently no widely used tool for measuring whether services are working in recovery orientated ways. Any of the above personal recovery measures would have to be contrasted with measures of clinical recovery such as the HoNOS (Health of the Nations Outcome Scale) which is well used in New Zealand.

### Norway

Recovery and perspectives of service users are given increasingly attention in Norwegian national health policies and partly in mental health services. But this development is still in an early phase compared to most other countries presented in this article. There have been no specific national programs on recovery. But elements of recovery-oriented practices to help the service user to live a normal life were among the aims of the National Plan for Mental Health 1999–2008 [43, 44]. However, the quantified goals in the plan were focusing on up-scaling service provision, and there were no measurements on quality of the mental health services. During the last years there has been increased emphasis on services user preferences and perspectives in national mental health policy. But it still remains to see a clear change in practice and culture throughout the mental health services and by the professional groups. However, focus on recovery has been emphasized by some university colleges and user organizations. The National Competence Centre for Mental Health care in the Municipalities and the National Centre for Knowledge through Experience in Mental Health are two centers that are funded by the national health authorities to disseminate knowledge and experiences, and this includes emphasis on recovery and on service user involvement and empowerment. Recent national guidelines on mental health and substance abuse care puts great emphasis on recovery-oriented practices and perspectives [45]. A comprehensive overview of recovery practices has also been published in Norwegian [46]. There are no published Norwegian measurements of recovery, but some questionnaires on recovery have been translated into Norwegian and are used in research projects, including the process of recovery questionnaire (QPR) [47] and the recovery assessment scale (RAS) [48].

### Scotland

The Scottish Recovery Network was established in 2004 and is part of the overall Scottish Mental Health Strategy laid out in several documents including *Scotland’s Mental Health Strategy: 2012*–*2015* [49] which builds on previous documents *Delivering for Mental Health* [50] and *Towards a Mentally Flourishing Scotland* [51]. The strategy identifies *seven themes* that cut across the mental health work program for promotion, prevention, treatment, care and recovery (e.g., working with families and carers; peer work and support; support for self-management and self-help; stigma and right of people with mental illness, personal, social, and clinical outcomes; and efficient use of new technology). There are currently no mandatory, universal clinical outcome measures in mental health. Ongoing work on a mental health quality indicator profile has recommended that there is a role out nationally, across mental health services, of the recording and reporting of a balanced set of measures that span the dimensions of Scotland’s quality strategy for mental health services—timely, equitable, effective, efficient, person centred and safe (five measures of each are in development).Patient safety experience is also measured through the National availability of a patient safety climate tool developed through the Scottish Patient Safety Programme. Core Net is currently used in several services as a clinical outcome tool allowing individual recovery monitoring and service level effectiveness measurement.

The Scottish Recovery Indicator 2 (SRI 2) framework, [52] designed by the Scottish Recovery Network, was launched at the end of October 2011 and constitutes a revised and enhanced version of the first 2009 Scottish Recovery Indicator (SRI). This is a service development tool for mental health to develop a cycle of continuous improvement. It is based on ten recovery indicators and six sources of information (assessments, care plans, service information, service provider, service user, and informal carer). It aims to support services and teams to enhance their recovery approach through self-assessment. As of August 2013, SRI 2 had been completed to action planning stage by over 270 services across Scotland.

The voluntary sector have been using recovery outcome focused measurements such as the Recovery Star and these approaches have increasingly been used in conjunction with statutory services in collaborative care approaches. The Individual Recovery Outcomes Counter tool (I.ROC) has been developed by a third sector voluntary organization. Wellness Recovery Actions Plans and Advanced statements are other examples of personalized recovery supporting documents.

### United States

The concepts of recovery and recovery-oriented services and systems are increasingly integrated into behavioral health care in the United States. In 2011, the Substance Abuse and Mental Health Services Administration (SAMHSA) identified “Recovery Support” as one of its eight Strategic Initiatives and launched its Recovery Support Strategic Initiative. Through the Recovery Support Strategic Initiative, the agency has delineated four major dimensions that support a life in recovery—*Health, Home, Purpose,* and *Community*. In addition, SAMHSA established 10 guiding principles [53] of recovery and recovery oriented services which include the following components: hope, person-driven, many pathways, holistic, peer-support, relational, culture, addresses trauma, strengths/responsibility, and respect.

SAMHSA has also been pilot-testing a recovery measurement tool comprised of 21 questions that explore a patient’s quality of life and overall health. The SAMHSA tool includes the World Health Organization Quality of Life (QOL)-8 as well as a set of additional questions designed to specifically learn about the individual’s mental health and substance abuse recovery and sense of efficacy in navigating the healthcare system and managing the full gamut of housing, relationships, and wellness. Upon successful completion of the pilot test, the recovery tool will be embedded in SAMHSA’s data collection efforts across the United States.

At the same time, SAMHSA launched BRSS TACS (Bringing Recovery Supports to Scale Technical Assistance Center Strategy), a 5-year national training and technical assistance project [54] that supports the expansion and integration of recovery-oriented care delivered by mental health providers. Within the context of health reform and drawing on research, practice, and personal experience of recovering individuals, SAMHSA is leading efforts to advance the understanding of recovery and ensure that recovery-oriented behavioral health services and systems are adopted and implemented in every state and community.

## Discussion

Participating countries are at various stages of developing and implementing recovery frameworks and measures for routine use in mental health care. Some countries have developed their own set of recovery measures (e.g., Scotland) while others seek to adapt measures developed by other countries to their particular systems and identified needs (e.g., Australia, New Zealand). A recovery *orientation* has been embraced as a central component of mental health policy in most Anglophone countries (Australia, Canada, England, New Zealand, Scotland, and US) and recently also in the Netherlands. However, countries’ programs described here are not necessarily based on the same conceptual understanding of recovery and the degree to which mental health services are or should be penetrated by this approach. Despite growing acknowledgement of service users’ role in the decision-making process of what kind of services they are receiving, most mental health systems are still far from implementing recovery-oriented practices across all services. Some countries are just at the beginning of this process while others have already invested heavily in bringing their mental health system in line with recovery- oriented practices including the development and implementation of recovery-oriented measures as part of routine quality measurement in mental health care. The programs and initiatives described here need to be contextualized not only within the legal and structural framework of a country’s health system, but also within the wider national context of work undertaken in various areas and jurisdictions of each country: e.g., changes in legislation, the development of a national framework and increased research opportunities and funding are important preconditions to pave the way for a paradigm shift within a country’s mental health care system. Equally important, however, seems to be a buy-in from major stakeholders—i.e., providers and payers/funders of mental health services and service user groups. The level of involvement and degree of political activity that the latter can mount to shape strategies for mental health care reform seem to be another important supporting element for moving towards recovery-oriented services across the system. Another important factor is the education of mental health professionals, service users and their families and the public at large to achieve an overall culture change regarding mental illness. National health care systems like Scotland’s seem to facilitate the channeling of these different components into one cohesive development and implementation strategy as demonstrated in the development and implementation of SRI.

This review provides a point-in time overview of where countries are in terms of prioritizing recovery-oriented services and how they approach implementing services that align with the principles of recovery-oriented mental health care. To move from commitment statements and frameworks to the actual implementation of recovery principles across all services and levels of care, it will be important to build on this collective knowledge and use the emerging research to shape and inform future policies. This will require not only commitment at the policy and service provider level on the one hand but renewed commitment by the research community as well. In addition, the field needs a better understanding of the relationship between recovery outcomes and traditional clinical outcomes, and how to better operationalize service user reported outcomes measures as well as those measures that are aimed at the recovery orientation of service providers or systems. Frameworks like CHIME can provide a taxonomy of a common recovery outcomes framework and support the development of measures of recovery, both at the personal and system level.

## Conclusions

This overview provides insights into the current state of recovery-oriented mental health care and countries’ different approaches for implementing recovery oriented services and measures. While recovery has gained acceptance as an important domain in health care in many countries, the implementation and evaluation of recovery concepts throughout care delivery systems in an ongoing and consistent way is still a work in progress and will need sustained resources and commitment by all stakeholders involved in this process.

## Abbreviations

ADOM: alcohol and drug outcome measure
ARI: advancing recovery in Ireland
AWMF: German Association of the Scientific Medical Societies
BRSS TACS: Bringing Recovery Supports to Scale Technical Assistance Center Strategy
DGPPN: German Association for Psychiatry, Psychotherapy and Psychosomatics
HoNOS: Health of the Nations Outcome Scale
HRB: Health Research Board (Ireland)
IIMHL: International Initiative for Mental Health Leadership
I.ROC: Individual Recovery Outcomes Counter tool
MANSA: Manchester Short Assessment of Quality of Life
MHCC: Mental Health Commission of Canada
QOL: quality of life
QPR: the process of recovery questionnaire
QUARTS: quality assessment of regional treatment systems for schizophrenia
RAS: the recovery assessment scale
ROPI: recovery oriented practices index
SAMHSA: Substance Abuse and Mental Health Services Administration
SMI: severe mental illness
SRI: Scottish recovery indicator
